# Estimation of the variations in mechanical impedance between the actuator and the chest, and the power delivered to the chest during cardiopulmonary resuscitation using machine-embedded sensors

**DOI:** 10.1186/s12938-018-0521-5

**Published:** 2018-06-19

**Authors:** Seong Wook Choi, Do Yeon Lee, Kyoung Won Nam

**Affiliations:** 1 Program of Mechanical & Biomedical Engineering, Kangwon National University, Chuncheon, South Korea; 2 Division of Biomedical Engineering, Pusan National University Yangsan Hospital, Yangsan, South Korea; 3 Department of Biomedical Engineering, School of Medicine, Pusan National University, Yangsan, South Korea

**Keywords:** Mechanical impedance, Cardiopulmonary resuscitation machine, Chest compression, Real-time frequency analysis, Chest model

## Abstract

**Background:**

To reduce the risk of patient damage and complications during the cardiopulmonary resuscitation (CPR) process in emergency situations, it is necessary to monitor the status of the patient and the quality of CPR while CPR processing without additional bio-signal measurement devices. In this study, an algorithm is proposed to estimate the mechanical impedance (MI) between an actuator of the CPR machine and the chest of the patient, and to estimate the power delivered to the chest of the patient during the CPR process.

**Methods:**

Two sensors for force and depth measurement were embedded into a custom-made CPR machine and the algorithm for MI and power estimation was implemented. The performance of the algorithm was evaluated by comparing the results from the kinetic model, the conventional discrete Fourier transform (DFT), and the proposed method.

**Results:**

The estimations of the proposed method showed similar increasing/decreasing trends with the calculations from the kinetic model. In addition, the proposed method showed statistically equivalent performance in the MI estimation, and at the same time, showed statistically superior performance in the power estimation compared with the calculations from the conventional DFT. Furthermore, the MI and power estimation could be performed almost in real-time during the CPR process without excessive hands-off periods, and the intensity of random noise contained in the input signals did not seriously affect the MI and power estimations of the proposed method.

**Conclusion:**

We expect that the proposed algorithm can reduce various CPR-related complications and improve patient safety.

## Background

When emergency situations occur that induce stopping of the heart (e.g., drowning or cardiac arrest), it is important to perform the correct cardiopulmonary resuscitation (CPR) process as soon as possible to recover the autonomic beating of the native heart and prevent serious brain damage [1]. When such an emergency situation occurs in locations outside of a hospital due to accidents or disease, emergency services should be immediately contacted and manual CPR process needs to be performed repetitively until the arrival of the trained paramedics. The paramedics then consistently perform emergency rescue processes using an automated external defibrillator and a CPR machine while transporting the patient to the hospital [2, 3]. The CPR machine delivers repetitively short and strong pressing action to the patient’s chest via an actuator. However, when the power is too high, various injuries can occur such as ecchymosis in the tissues and organs, myocardial rupture, and laceration [4, 5]. In addition, when the CPR position is changed during the process due to the wobble or vibration of the ambulance while transporting, the efficacy of the CPR process deteriorates and additional harm to the patient can occur [6]. To reduce such risk of patient harm, it is necessary to consistently monitor the status of patient and the quality of CPR during the operation of the CPR machine [3].

The most representative method for this purpose, recommended by the American Heart Association (AHA), is capnography. Capnography measures the end-tidal carbon dioxide (ETCO_2_) through endotracheal intubation, which is closely related to myocardial blood flow [3, 7]; however, its application in emergency situations is limited due to the risk of the improper insertion of an intubation tube and accidental displacement of the tube during transport [8, 9]. Currently, some of the automated external defibrillators utilize the electrocardiogram (ECG) signals of the patient to determine the need for further electric shock. However, for recording and investigating the ECG signals, hands-off intervals without chest compression are required, which can reduce the return of spontaneous circulation (ROSC) rate [10, 11]. In other studies, various physiological signals, such as blood flow (BF), blood pressure (BP), and electroencephalogram (EEG), have also been utilized to assess the status of the patient and the quality of the CPR process [12–14]. However, such bio-signal-based methodologies have several limitations: (1) they are only applicable when the relevant sensors and measurement devices have already been applied to the patient; (2) the variation in skin–electrode impedance affects the quality of the measured signals; and (3) bio-signal measurement is unavailable during the CPR process due to motion artifact. Until now, to the best of our knowledge, no studies have been performed that aim to consistently monitor the status of the patient and the quality of CPR during the CPR process. Therefore, considering the importance of patient safety in emergency situations, a new technique for such purpose is required.

In this study, an algorithm was proposed to estimate the mechanical impedance (MI) between the actuator of the CPR machine and the chest of the patient, and at the same time, to estimate the power delivered to the chest of the patient during the CPR process.

## Methods

### Configuration of the utilized CPR machine

**Fig. 1:**
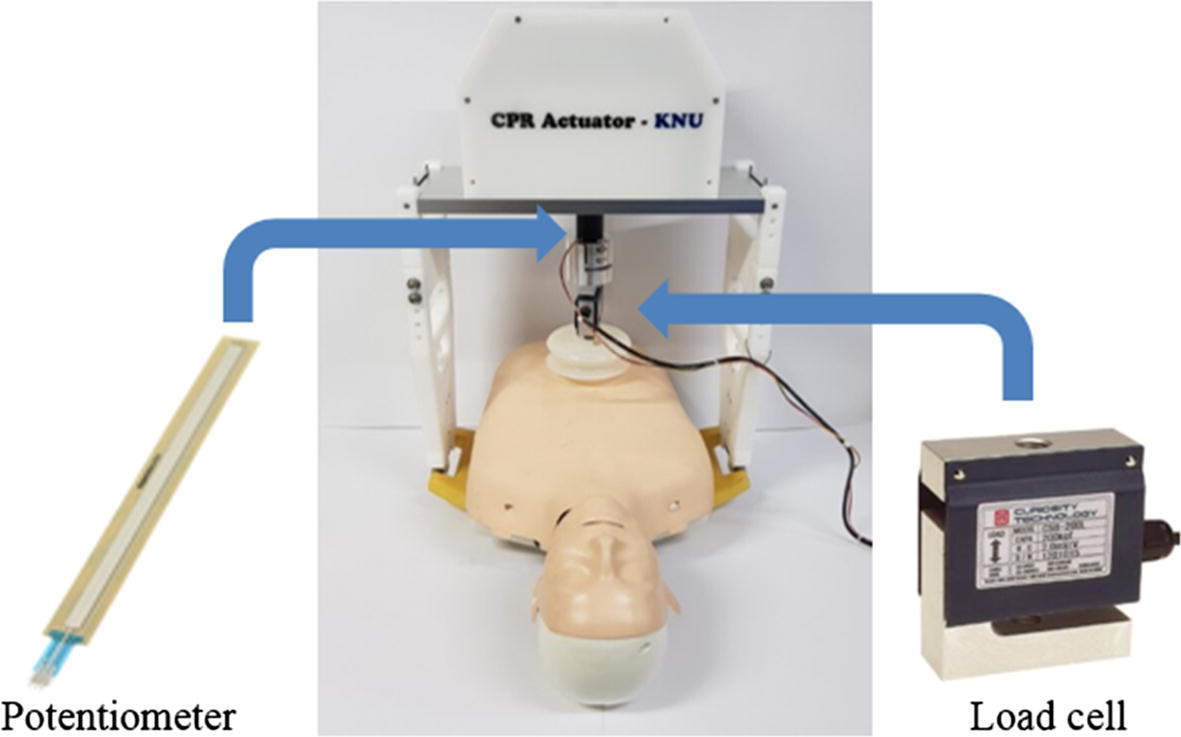
CPR assist device utilized in this study. A potentiometer and a load cell were embedded in the device to measure the variations in force and depth during the CPR process

To measure the MI between the actuator and the chest, information about the magnitude of the chest-compression force and the chest-compression depth during CPR process is necessary. In this study, a load cell (CSB-200L; Curiotec Co. Ltd., Paju, Korea) and a potentiometer (SPL0170103ST; Spectra Symbol Corp., Salt Lake, USA) were embedded in a custom-made CPR machine (Fig. 1). The operation ranges of the utilized CPR machine were a compression depth of 3–5.5 cm, a compression rate of 80–110 compressions per minute (cpm), and a maximal compression force of 60 Kgf by referring to the recommendations of the AHA (compression depth of 5 cm with above 100 cpm) [15–17]. The ratio between compression/decompression times was fixed to 40%, and the measured force and depth signals were digitized at a sampling rate of 100 Hz with a 10 bit resolution.

### Proposed algorithm to estimate the MI and power during the CPR process

To estimate the MI using the sensor measurements, the number of input data during one period of chest compression (*N*) was first obtained as the number of data between two adjacent peaks in the measurements of the chest-compression force. From the measurements of the load cell and the potentiometer, the magnitude and the phase of the force (*F*) and the depth (*D*) for one chest-compression period were calculated using Eqs. (1)–(5) based on the discrete Fourier transform (DFT) [18, 19].

1 $$\left| F(\omega) \right|\,=\,\frac{2}{N}\sqrt{{{\left(\sum\limits_{n\,=\,n\,-\,N\,+\,1}^{n}{F(n)\cos \left(\frac{2\pi n}{N} \right)} \right)}^{2}}\,+\,{{\left(\sum\limits_{n\,=\,n\,-\,N\,+\,1}^{n}{F(n)\sin \left(\frac{2\pi n}{N} \right)} \right)}^{2}}}$$

2 $$\angle F(\omega)\,=\,{{\tan}^{-1}}\left(\frac{\sum\nolimits_{n\,=\,n\,-\,N\,+\,1}^{n}{F(n)\sin \left({}^{2\pi n}/{}_{N} \right)}}{\sum\nolimits_{n\,=\,n\,-\,N\,+\,1}^{n}{F(n)\cos \left({}^{2\pi n}/{}_{N} \right)}} \right)$$

3 $$\left| D(\omega) \right|\,=\,\frac{2}{N}\sqrt{{{\left(\sum\limits_{n\,=\,n\,-\,N\,+\,1}^{n}{D(n)\cos \left(\frac{2\pi n}{N} \right)} \right)}^{2}}\,+\,{{\left(\sum\limits_{n\,=\,n\,-\,N\,+\,1}^{n}{D(n)\sin \left(\frac{2\pi n}{N} \right)} \right)}^{2}}}$$

4 $$\angle D(\omega)\,=\,{{\tan}^{-1}}\left(\frac{\sum\nolimits_{n\,=\,n\,-\,N\,+\,1}^{n}{D(n)\sin \left({}^{2\pi n}/{}_{N} \right)}}{\sum\nolimits_{n\,=\,n\,-\,N\,+\,1}^{n}{D(n)\cos \left({}^{2\pi n}/{}_{N} \right)}} \right)$$

5 $$\omega\,=\,\frac{2\cdot \pi}{N/{{f}_{S}}}$$

where *ω* represents the angular frequency and *f*_*s*_ represents the sampling frequency (fixed to 100 Hz). The magnitude of thoracic impedance is the ratio between the force of chest compression and the velocity of chest compression. When the variations in the depth of chest compression is represented as *D*(*ω*)*·sin*(*ωt *+ *ф*), the velocity of chest compression is calculated as *dD*(*t*)*/dt* = *ω·D*(*ω*)*·cos*(*ωt *+ *ф*), the frequency component of the velocity of chest compression is calculated as *ω·D*(*ω*), and the phase moves 90°. Therefore, the magnitude and the phase of the MI (*Z*) were calculated from Eqs. (6) and (7). The phases of *F*(*ω*) and *D*(*ω*) differ from the actual phases due to the time-delay from the peak detection process; however, because the phase of the MI can be obtained from the difference between *F*(*ω*) and *D*(*ω*), the time-delay from the peak detection process does not affect the phase of the MI, as shown in Eq. (7).

6 $$\left| Z(\omega) \right|\,=\,\frac{\left| F(\omega) \right|}{\omega \left| D(\omega) \right|}$$

7 $$\angle Z(\omega)\,=\,\angle F(\omega)\,-\,\left(\angle D(\omega)\,+\,{{90}^{\circ}} \right).$$

The power delivered to the patient’s chest by the CPR machine can be calculated as the multiplication of the force of chest compression and the velocity of chest compression. Therefore, the power of chest compression can be calculated as *F*(*ω*)*·sin*(*ωt *+ *θ*)*·ω·D*(*ω*)*·cos*(*ωt *+ *ф*)=(*ω·D*(*ω*)*·F*(*ω*)*/2*)*·*(*sin*(*2ωt *+ *θ*+*ф*)+ *sin*(*θ *− *ф*)), and finally, the magnitude of power delivered to the chest during the CPR process is calculated from Eq. (8).

8 $$\left| {{P}_{a}}(\omega) \right|\,=\,\frac{\omega \left| F(\omega) \right|\left| D(\omega) \right|}{2}$$

where *P*_*a*_ represents the magnitude of apparent power delivered to the chest, which induces mandatory blood circulation while the native heart is in fibrillation state.

### Evaluation of the performance of the proposed algorithm

In this study, we measured the magnitudes of the force of chest compression and the depth of chest compression during the CPR process using a commercialized dummy (Little Anne; Laerdal Medical AS, Stavanger, Norway) which contained internal springs that can reproduce the chest-compression force while general CPR conditions. Three springs with different spring constants that can be installed inside the dummy were utilized, and the values of the calculated elastic modulus (*k*_*e*_) of the dummy were 680, 890, and 942 Kgf/m for each spring, respectively, by referring to the *k*_*e*_ values in the study of Arbogast et al. [20].

First, to evaluate the accuracy of the proposed method, the actual values of the MI and the power during the CPR process should be measured using appropriate sensing devices. Also, the measurements with the estimated values for the same operating conditions need to be compared. However, we could not find appropriate real-time sensing devices for this purpose. As an alternative, we evaluated the accuracy of the proposed algorithm indirectly using a conventional DFT as follows. (1) The rate and the depth of the chest compression were fixed at 100 cpm and 5 cm, respectively. (2) The CPR machine was operated for 90 s and the measurements of the embedded sensors were then acquired (in the current implementation, one MI data and one power data are acquired per each compression period; i.e., 152 MI and 152 power data are acquired from the CPR machine test because the CPR machine compresses the chest 152 times during 90 s when the rate of chest compression is fixed at 100 cpm). (3) The impedance at 100 cpm was calculated using the DFT of the force and velocity per every 3 s, and the velocity was also calculated by multiplying the angular frequency *ω* and the DFT of the measured chest-compression depth. (4) The power was calculated by averaging the multiplication of the measured force and the calculated velocity for each compression period (one MI data and one power data are acquired per each DFT; i.e., 30 MI and 30 power are acquired from the conventional DFT because each DFT process uses 3 s data) [18, 19]. (5) Steps 2–4 were repeated for each of the three springs. In addition, as a reference, a kinetic model of the chest-compression force [*Force *= elastic modulus * Depth] was utilized. Then, the statistical significance of the differences between the calculations from conventional DFT (steps 1–5 above) and the estimations from the proposed method [Eqs. (6) and (8)] was evaluated using the Levene’s test.

Second, to verify the effect of variations in the rate and the depth of chest compression during the CPR process on the MI and power estimation, the implemented CPR machine was installed on the dummy (Fig. 1), and the CPR machine test was performed as follows: (1) P1: the rate of chest compression was fixed at 100 cpm, the value of *k*_*e*_ of the spring was fixed at 680 Kgf/m, and the depth of chest compression varied at 3, 4, and 5 cm with 1 cm step. (2) P2: the depth of chest compression was fixed at 5 cm, the value of *k*_*e*_ of the spring was fixed at 680 Kgf/m, and the rate of chest compression varied at 80, 90, 100, and 110 cpm (Table 1). Then, the same patterns of chest compression (P1 and P2) were also applied to the kinetic model, and the results from the kinetic model investigation and the CPR machine test were compared with each other.

**Table 1:** Parameter variations in the CPR machine test

Test pattern	CPR machine test		
	P1	P2	P3
Depth of chest compression (cm)	5	3/4/5	5
Rate of chest compression (cpm)	100	100	80/90/100/110
Elastic modulus of spring (Kgf/m)	680/890/942	680	680

Third, to observe the effect of variations in the value of *k*_*e*_ of the spring on the MI and power estimation, an additional CPR machine test was performed as follows: (3) P3: the rate of chest compression was fixed at 100 cpm, the depth of chest compression was fixed at 5 cm, and the value of *k*_*e*_ of the spring inside the dummy varied at 680, 890, and 942 Kgf/m. Then, the same patterns of chest compression (P3) were also applied to the kinetic model and the conventional DFT, and the results from the three methodologies were compared with each other.

During the CPR machine test (P1, P2 and P3), 152 MI data and 152 power data were acquired from the measurements for each test condition, and during the conventional DFT analysis, 30 MI data and 30 power data were acquired from the measurements for each test condition, respectively.

**Fig. 2:**
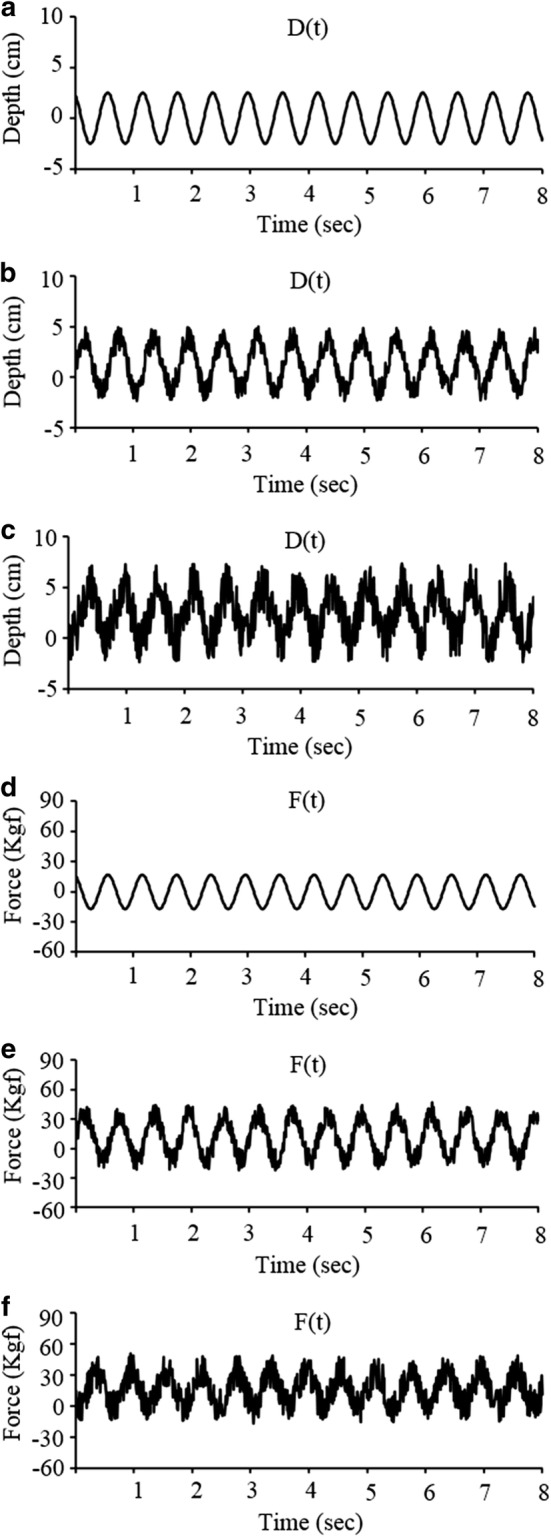
Simulated depth (*D*) and force (*F*) signals that are mixed with random noise for the noise-tolerance test of the proposed method. **a** Clean *D*(*t*), **b** Clean *F*(*t*), **c** *D*(*t*) with 6 dB SNR **d** *F*(*t*) with 6 dB SNR **e** *D*(*t*) with 1.5 dB SNR **f** *F*(*t*) with 1.5 dB SNR

Fourth, to evaluate the effect of variations in the intensity of noises contained in the *D*(*t*) and *F*(*t*) signals on the MI and power estimation, two random noises, of which the variance is 16.7% (signal-to-noise ratio [SNR] = 6 dB) and 66.7% (SNR = 1.5 dB) of that of the *D*(*t*) and *F*(*t*) signals, were mixed with the clean *D*(*t*) and *F*(*t*) signals, and those noise-contaminated signals were applied to the proposed method (Fig. 2). The magnitude of the depth of the chest compression was fixed at 5 cm, the rate of chest compression was fixed at 100 cpm, and the value of *k*_*e*_ was set at 680, 890, and 942 Kgf/m.

## Results

**Fig. 3:**
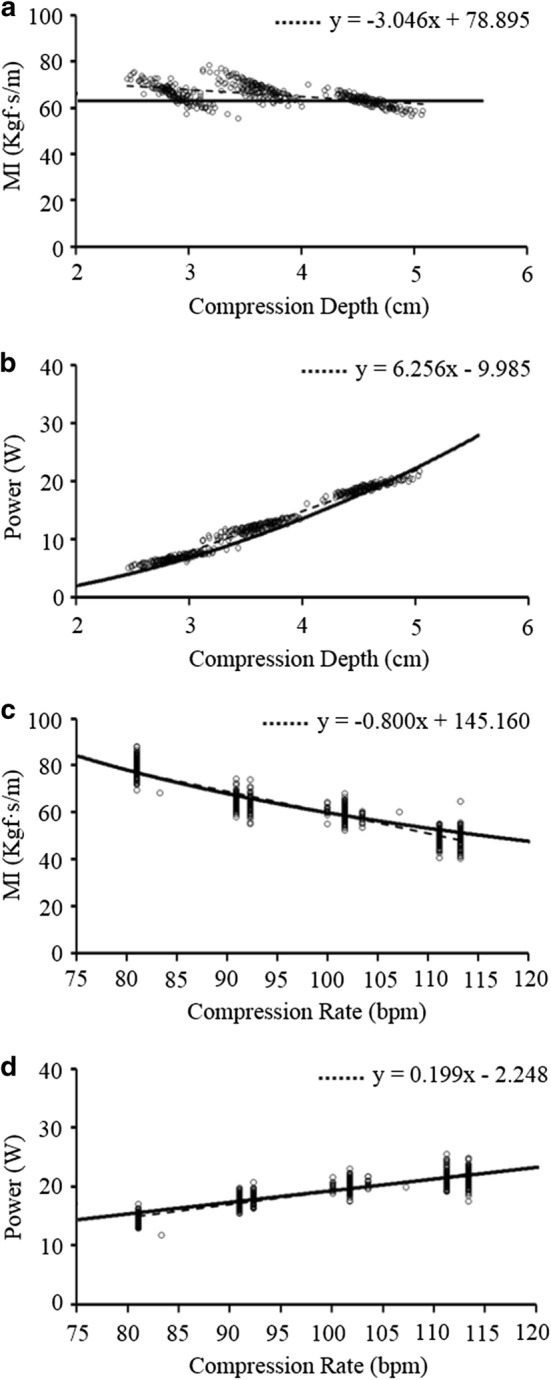
Results of the comparison between the kinetic model and the proposed method in P1/P2 conditions (N = 152 for each condition). Solid lines represent the results of the kinetic model investigation and dashed lines represent the results of the proposed method (linear regression). *MI* mechanical impedance. **a** Magnitude of the MI in P1 condition, **b** magnitude of the power delivered to the chest in P1 condition, **c** magnitude of the MI in P2 condition, and **d** magnitude of the power delivered to the chest in P2 condition

Figure 3 presents a comparison between the results from the kinetic model and those from the proposed method (i.e., CPR machine test). For the P1 condition, the magnitude of the MI was fixed at a constant value (64.9 Kgf s/m) regardless of the variations in the depth of chest compression in the kinetic model, and was 68.1 ± 3.8, 70.4 ± 3.4, and 64.8 ± 2.5 Kgf s/m when the depth of chest compression was 3, 4, and 5 cm, respectively, in the proposed method (Fig. 3a). The magnitude of the power increased as the depth of the chest compression increased in the kinetic model, and also increased to 7.65 ± 0.6, 12.4 ± 0.8, and 18.4 ± 0.8 Kgf s/m when the depth of the chest compression was 3, 4, and 5 cm, respectively, in the proposed method (Fig. 3b). For the P2 condition, the magnitude of the MI decreased as the rate of the chest compression increased in the kinetic model, and also decreased to 82.1 ± 3.2, 69.1 ± 3.0, 64.7 ± 2.5, and 55.6 ± 3.1 Kgf s/m when the rate of the chest compression was 80, 90, 100, and 110 cpm, respectively, in the proposed method (Fig. 3c). The magnitude of the power increased as the rate of the chest compression increased in the kinetic model, and also increased to 13.4 ± 0.7, 16.3 ± 0.9, 18.5 ± 0.8, and 19.7 ± 1.2 Kgf s/m when the rate of the chest compression was 80, 90, 100, and 110 cpm, respectively, in the proposed method (Fig. 3d).

**Fig. 4:**
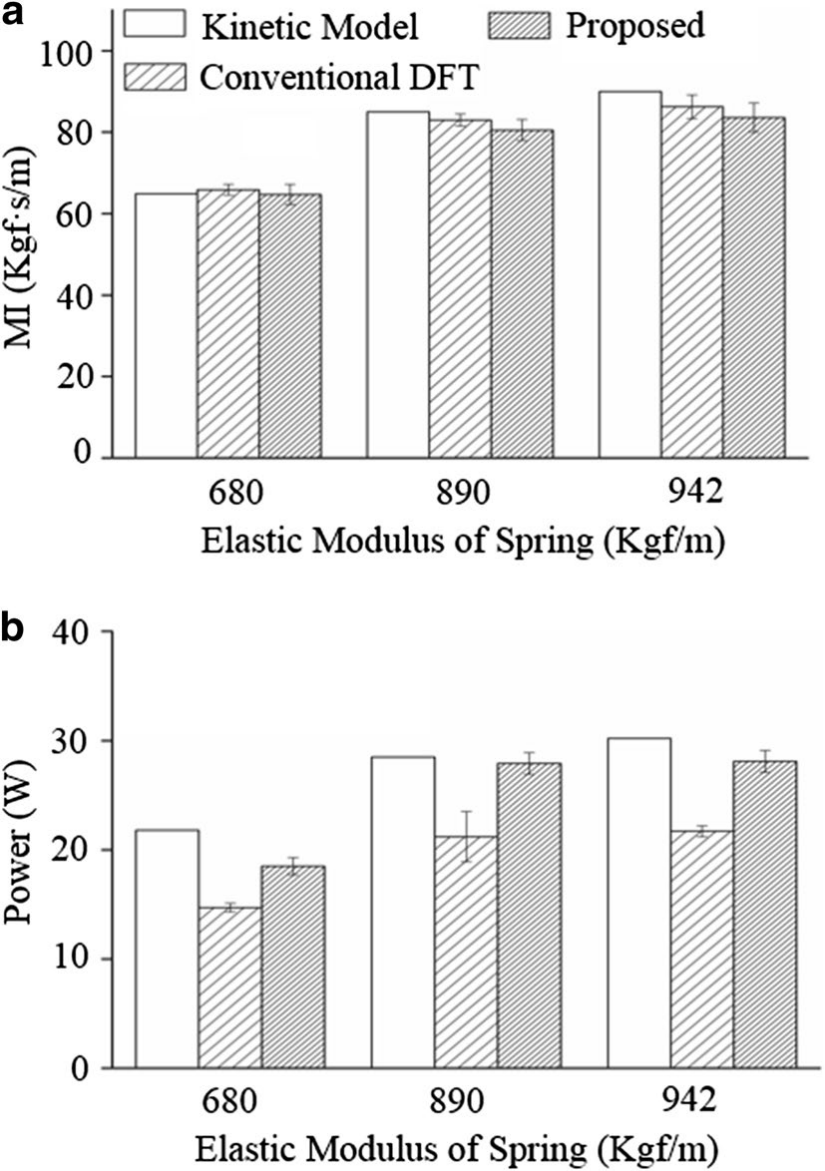
Results of the comparison among the three applied methodologies in P3 condition. N = 30 for the conventional DFT and N = 152 for the proposed method. **a** MI, **b** power delivered to the chest. *MI* mechanical impedance

Figure 4 presents a comparison among the three applied methodologies—i.e., the kinetic model, the conventional DFP, and the proposed method—at P3 condition. When *k*_*e*_ = 680 Kgf/m, the magnitude of MI was 64.9, 65.9 ± 1.3, and 64.7 ± 2.5 Kgf s/m in the kinetic model, the conventional DFT, and the proposed method; in addition, the magnitude of the power delivered to the chest was 21.8, 14.7 ± 0.4, and 18.5 ± 0.8 W in the same tests. When *k*_*e*_ = 890 Kgf/m, the magnitude of MI was 85.0, 83.0 ± 1.5, and 80.5 ± 2.6 Kgf s/m in the kinetic model, the conventional DFT, and the proposed method; in addition, the magnitude of the power delivered to the chest was 28.5, 21.2 ± 2.6, and 27.9 ± 1.0 W in the same tests. When *k*_*e*_ = 942 Kgf/m, the magnitude of MI was 90.0, 86.3 ± 2.9, and 83.6 ± 3.6 Kgf s/m in the kinetic model, the conventional DFT, and the proposed method; in addition, the magnitude of the power delivered to the chest was 30.2, 21.7 ± 0.5, and 28.1 ± 1.0 W in the same tests. Absolute errors (and error-rates) in the MI between the kinetic model and the conventional DFT were 1.0 Kgf s/m (1.5%), 2.0 Kgf s/m (2.4%), and 3.7 Kgf s/m (4.1%) on average when the value of *k*_*e*_ was 680, 890, and 942 Kgf/m, respectively; in contrast, those between the kinetic model and the proposed method were 0.2 Kgf s/m (0.3%), 4.5 Kgf s/m (5.3%), and 6.4 Kgf s/m (7.1%), respectively, on average. In addition, absolute errors (and error-rates) in the power between the kinetic model and the conventional DFT were 7.1 Kgf s/m (32.6%), 7.3 Kgf s/m (25.6%), and 8.5 Kgf s/m (28.1%) on average when the value of *k*_*e*_ was 680, 890, and 942 Kgf/m, respectively; in contrast, those between the kinetic model and the proposed method were 3.3 Kgf s/m (15.1%), 0.6 Kgf s/m (2.1%), and 2.1 Kgf s/m (7.0%), respectively, on average. In Levene’s test, there was no significant difference in the MI between the values from the conventional DFT and the proposed method; in contrast, there was significant difference in the power delivered to the chest between the two methodologies, which demonstrates that the accuracy of the proposed method is superior to that of the conventional DFT in estimating the power delivered to the chest during the CPR process.

**Fig. 5:**
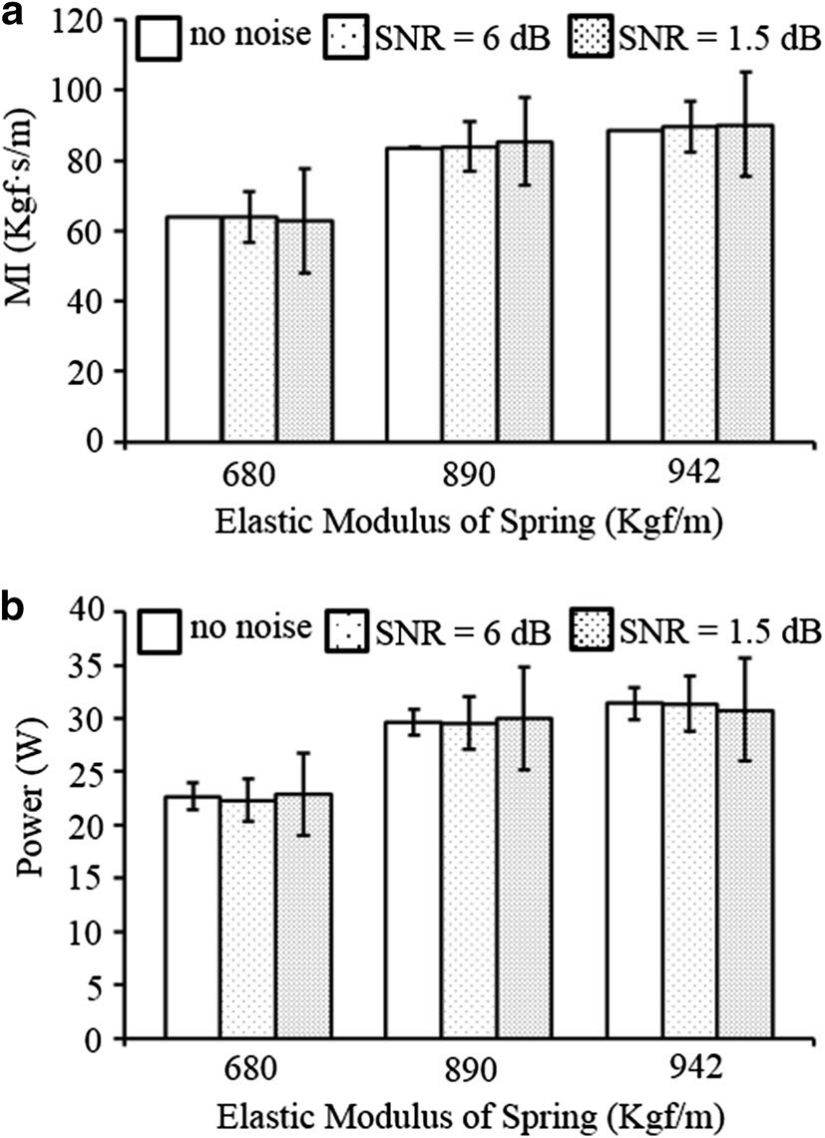
Results of the comparison test carried out to verify the effects of the noise intensities in force and depth signals on the estimation of MI in the proposed method with various *k*_*e*_ values. *MI* mechanical impedance. **a** MI, **b** power delivered to the chest

Figure 5 shows the effects of the noise intensities on the force and depth signals on the estimation of MI and power in the proposed method with various *k*_*e*_ values. When *k*_*e*_ = 680 Kgf/m, the magnitudes of MI and power were 68.9 ± 0.1 Kgf s/m and 22.7 ± 1.1 W for clean signals, respectively, 64.6 ± 4.8 Kgf s/m and 22.1 ± 2.0 W for noisy signals with 6 dB SNR, respectively, and 65.4 ± 10.5 Kgf s/m and 22.8 ± 3.9 W for noisy signals with 1.5 dB SNR, respectively. When *k*_*e*_ = 890 Kgf/m, the magnitudes of MI and power were 83.6 ± 0.2 Kgf s/m and 29.7 ± 1.5 W for clean signals, respectively, 84.0 ± 6.3 Kgf s/m and 29.4 ± 2.4 W for noisy signals with 6 dB SNR, respectively, and 84.4 ± 13.6 Kgf s/m and 28.6 ± 4.5 W for noisy signals with 1.5 dB SNR, respectively. When *k*_*e*_ = 942 Kgf/m, the magnitudes of MI and power were 88.4 ± 0.1 Kgf s/m and 31.4 ± 1.6 W for clean signals, respectively, 88.5 ± 7.1 Kgf s/m and 31.2 ± 2.6 W for noisy signals with 6 dB SNR, respectively, and 87.4 ± 13.1 Kgf s/m and 31.0 ± 5.8 W for noisy signals with 1.5 dB SNR, respectively.

## Discussion

Maltese et al. proposed a lumped parameter model of the thoracic cavity based on the external power, compression depth, and compression velocity during the CPR process [21]. In their model, the MI could be calculated based on the elastic coefficient (*k*_*e*_) and the viscous coefficient (*μ*) as $MI\,=\,\frac{(\mu \, \times \, j\frac{{{k}_{e}}}{2\pi f})}{(\mu \,+\, j\frac{{{k}_{e}}}{2\pi f})}$. In addition, Arbogast et al. compared thoracic compression response during CPR process between living and post-mortem human subjects using a load cell and an accelerometer, where data were modeled with a progressive spring in parallel with a viscous damper [20]; in their study, the *k*_*e*_ value of the chest of post-mortem human subjects increased 182% compared to that of a living body, and the *μ* value decreased 60%, respectively. These results demonstrate that the measurements of MI (affected by *k*_*e*_ and *μ*) can reflect the physiological status of a patient; e.g., dead or alive. However, in their study, measurements of *k*_*e*_ and *μ* requested additional dedicated devices and the measured data were prone to noise-contaminated; therefore, the method is not appropriate for real-time CPR-assistance purpose. In contrast, in our proposed method, the MI can be calculated based only on the measurements of CPR machine-embedded sensors in real-time, which is more suitable for CPR-assistance purpose. In addition, when the patient’s heart is stopped, the power delivered to the chest by the CPR machine is the sole energy source that induces blood circulation in the body; therefore, the magnitude of the power delivered to the chest, as well as compression rate and compression depth, can be related to the quality of CPR, although more investigations are required to further verify the direct relationship between the two.

In Fig. 3, the estimations of the proposed method (dashed lines) showed similar increasing/decreasing trends with the calculations from the kinetic model (solid lines). In addition, in Fig. 4, the proposed method showed statistically equivalent performance (error rates of 2.4–4.2% at the conventional DFT and 0.9–6.8% at the proposed method) in the MI estimation, and at the same time, showed statistically superior performance (error rates of 13.8–28.2% at the conventional DFT and 2.1–10.1% at the proposed method) in the power estimation compared with the calculations from the conventional DFT, which demonstrates the validity of the proposed method. Furthermore, in Fig. 5, the intensity of random noise contained in the input signals did not seriously affect the estimations of MI and power values, regardless of the variations in elastic modulus. This noise-tolerance of the proposed method is based on the DFT utilized in the force and depth estimation; i.e., the magnitude of impulse noises reduces to 1/N of the original noise during the DFT for N data samples.

In most previous studies, the quality of CPR was evaluated using the measurements of various physiological signals (e.g., ECG, EEG, BP, and BF) that require complex and time-consuming installation processes such as electrode attachment and device set-up as well as hands-off periods to evade motion artifacts. This can result in a reduced ROSC rate [10, 11]. Capnography, which is the official recommendation of the AHA, has an advantage of continuously monitoring the status of a patient during the CPR process; however, complications related to the difficulty in tube insertion and tube-related accidents during patient transport have been consistently reported [8, 9, 22, 23]. In other reports, the authors insisted that capnography can only show trends, and cannot directly demonstrate the quality of CPR because it does not clearly change in accordance with the variations in the depth and the rate of chest compression [7, 24, 25]. As described above, the estimations of the MI and the power from the proposed method can give additional information about the physiological status of patient and the quality of CPR [20, 21]. In addition, such estimation processes can be performed almost in real-time during the CPR process without excessive hands-off periods; more specifically, the time-delay between the signal input and the MI and power calculation is about 0.6 s at 100 cpm. Considering these benefits, application of the proposed method together with other bio-signal measurement devices, such as capnography or ECG, will further improve patient safety in emergency situations, although several practical techniques should also be implemented in future studies.

To reduce the number of complications from the use of the CPR machine, various practical functions for patient safety improvement should be included in the embedded algorithm: (1) real-time detection of improper CPR position during CPR process (e.g., actuator slip to abdominal region by vibration or wobble while transporting) to prevent patient damage; (2) real-time detection of heart-beats during CPR operation to prevent excessive electric shocks; and (3) real-time detection of rib fracture during CPR process to prevent secondary damage to tissues and organs. With these functions, the CPR machine will be able to monitor the status of the patient and the quality of CPR in real-time and automatically adjust the internal control parameters to deal with unexpected emergency situations. Such capabilities are not available in most of the currently commercialized CPR machines. For example, Lucas-II™ (Physio-Control Inc., Lund, Sweden) compresses the center of chest for CPR, but several reports commented side-effects of the device when the position of compression is inappropriate [3, 6, 13, 26, 27]; and AutoPulse™ (Zoll Medical Corp., Chelmsford, USA) compresses overall chest area for CPR, but several reports commented the risk of damage in other organs during CPR process [2, 3, 7, 26, 27]. If the currently proposed real-time MI and power estimation algorithm is applied to the control of the CPR machine and more dedicated safety functions, as described above, are realized later, complications related to the CPR machine can be reduced because the MI variations would reflect the variations in chest features such as thoracic skeleton, ligaments, and connected muscles, and the movements of the heart, lungs, and other organs during the CPR process. For example, if a rib fracture occurred or the actuator slipped into the abdomen region during the CPR process, the MI and power signals would rapidly show an abnormal pattern, within a short interval, that can be detected by the dedicated algorithm. In addition, we have a hypothesis that the mechanical properties of a blood-filled heart and an emptied heart would be different and this difference may be detected by applying more advanced signal investigation techniques, such as independent component analysis and machine learning, to the MI and power signals, which can be utilized to detect the heart-beat detection function during the CPR process; in fact, all of these exemplified safety functions are the topics of our subsequent study using animal experiments. Furthermore, from the implementation and evaluation of these protection algorithms in further studies, we expect it will be possible to develop a new CPR machine with closed-loop control scheme and enhanced patient safety.

The limitations of the current study are as following. (1) In this study, we selected the test conditions as 3–5 cm compression depth and 80–110 cpm compression rate to ease the data acquisition and processing. Although these ranges contain the representative recommendation of the AHA (5 cm with 100 cpm), in recent studies, higher compression rate (> 100 cpm) is generally emphasized for improved CPR efficiency; e.g., Alderman et al. recommended 120 cpm in their study [28]. (2) The kinetic model used in this study did not reflect the viscous coefficient of the real body, and so, calculations of the kinetic model did not reflect the actual cases accurately. To improve the accuracy of the model investigations, more realistic chest model reflecting both the elasticity and viscosity should be implemented in future study.

## Conclusion

The experimental results demonstrated the performance and noise-tolerance of the proposed method. We expect that the proposed method can reduce various CPR-related complications and improve patient safety.

## Abbreviations

CPR: cardiopulmonary resuscitation
MI: mechanical impedance
AHA: American Heart Association
ETCO_2_: end-tidal carbon dioxide
ECG: electrocardiogram
ROSC: return of spontaneous circulation
BF: blood flow
BP: blood pressure
EEG: electroencephalogram
SNR: signal-to-noise ratio
